# Strategies to Advance Women: Career Insights From Senior Leadership Women in Professional Sport in Canada

**DOI:** 10.3389/fspor.2021.716505

**Published:** 2021-09-13

**Authors:** Amanda Cosentino, W. James Weese, Janelle E. Wells

**Affiliations:** ^1^School of Kinesiology, Western University, London, ON, Canada; ^2^School of Marketing and Innovation, University of South Florida, Tampa, FL, United States

**Keywords:** women, sport leadership, sponsorship, career advancement, role model, mentorship

## Abstract

Women remain minimally represented in senior leadership roles in sport, despite increased female participation in both sport, sport management education programs, and in entry levels positions in the industry. Many women prematurely exit mid-level leadership positions in sport, or are often overlooked for senior leadership positions. To uncover the experiences and strategies of women who made it through the process, we interviewed all the women (*N* = 7) who now hold senior leadership positions with professional sport properties in Canada. Participants revealed they overcame real and perceived barriers, and they suggested women seeking senior leadership roles in the industry: (a) find, and later become role models, mentors, and sponsors; (b) create access to networks and opportunities; (c) strategically self-promote, and; (d) purposefully build a varied career portfolio. Recommendations for the industry and all those who work in the industry are presented with a goal to break the cycle and help ensure more equitable and inclusive leaders in the senior leadership ranks.

## Introduction

Diversity in organizations, and in particular, gender diversity has positive and tangible benefits to organizational performance, organizational culture, morale, and colleague motivation (Li and Nagar, 2013; Sheppard, 2018). This phenomenon is especially pronounced at the senior leadership ranks of organizations. For example, it has been uncovered that when women represent at least 30% of top management, organizations have been shown to perform significantly better than organizations without 30% of women in decision-making positions (Desvaux et al., 2007). Other researchers (Eagly, 2007; Johansen, 2007) have determined that when women are more proportionately represented in decision-making roles, their organizations have higher levels of performance, an enhanced public image, greater levels of employee satisfaction, higher levels of financial performance, and lower turnover levels. This may be due in part to women bringing diverse perspectives to decisions and/or contributing unique and creative ideas (Desvaux et al., 2007). Some researchers (Zenger and Folkman, 2012) go as far as suggesting that women are generally more effective leaders than men. Regardless of the reasons, it does not make sense to overlook 50% of the population for decision-making roles, especially in light of the purported benefits. However, this appears to be happening in sport, and especially at the senior leadership levels of the industry. The proportion of women in senior leadership roles (e.g., Vice President and above) is notably small and in some sports, non-existent (Dreher, 2003; Lough and Grappendorf, 2007; Sartore and Cunningham, 2007; Lapchick, 2016, 2020), in spite of the rising number of women playing sport (Burton, 2015) and/or enrolled in sport management educational programs (Moore et al., 2001; Spoor and Hoye, 2014). Calls for change are warranted.

The 2020 and 2021 Racial and Gender Report Cards published by The Institute for Diversity Ethics in Sport (2021) suggest little or no progress is being made in the United States professional leagues and the NCAA, in spite of government and institutional equity and diversity policies. However, although women remain vastly underrepresented in high-level leadership positions, participation rates have been rising at all levels of sport (i.e., interscholastic, intercollegiate, professional) in North America (Harris et al., 2015). According to data provided by both the National Centre for Education Statistics Report (2017); Statistics Canada Report Education in Canada: Key results from the 2016 census (2017), women are entering and graduating from North American universities at proportionally higher levels. Many women are attracted to sport management degree programs, and by extension, endeavor to secure leadership positions in the sport industry upon graduation (Harris et al., 2015; Hancock et al., 2018; Forsyth et al., 2019). One would assume that these factors, coupled with the billion-dollar growth of the sport's industry (Heitner, 2015) would translate to more senior leadership roles and specifically, more women occupying some of these roles. However, research has demonstrated that this transfer has not transpired, and especially not to the higher leadership echelons of organizations. Although women are making marginal gains in the number occupying senior leadership roles in Canadian professional sport (i.e., gain of 2% from 2017 to 2020), women still only occupy 14% of these senior roles. In addition, as of 2018, women only held 2% of the corporate board positions in professional sport. It appears as though the rungs of the corporate ladder for female sport leaders remain quite slippery. Why might this be the case?

Numerous researchers (Inglis et al., 2000; Hoeber and Frisby, 2001; Shaw and Hoeber, 2003; Shaw and Frisby, 2006; Hums and Yiamouyiannis, 2007; Moore et al., 2010; Burton et al., 2011; Masteralexis et al., 2011; Burton, 2015; Hancock and Hums, 2016; Wells and Kerwin, 2016; Burton and Leberman, 2017; Hartzell and Dixon, 2018; Darvin et al., 2019) have investigated the experience of female leaders at the coaching and administrative levels of sport, and have consistently found female representation to be low. With the notable exception of a few scholars (see Leberman and Palmer, 2009; Shaw and Leberman, 2015; Aman et al., 2018), limited to no researchers have explored the issue within Canada where many professional teams and leagues do not have a single woman in a senior leadership role. Although most of what our society understands about women sport leaders has been drawn from intercollegiate athletics settings (e.g., Burton, 2015; Wells et al., 2020), our research, like the research of Shaw and Leberman (2015) will be one of the few investigations to focus on senior women leaders' successful career advancement strategies. This study, set in Canadian professional sport landscape also responds to Hartzell and Dixon's (2018) call for sport management research set in different contexts. As such, the purpose of this study was 2-fold. First of all, this study was designed to explore the lived experiences of all women who currently hold senior leadership positions in professional sport in Canada. Secondly, researchers in this study gathered and deconstructed the insights, experiences, and suggestions of the women who advanced to these roles with hopes that their strategies would help other women progress to senior leadership roles in the industry. The research was guided by the following three research questions:

1. What are the career experiences of women senior leaders?

2. How, if at all, were women senior leaders supported in their pursuit of career advancement?

3. What do women senior leaders recommend the next generation of women do to advance their careers?

### Career Development Literature

The National Career Development Association (NCDA) defined career development as “the total constellation of psychological, sociological, educational, physical, economic, and chance factors that combine to influence the nature and significance of work in the total lifespan of any given individual” (National Career Development Association, 2003, p. 2). Researchers have explored the unique career paths of women in sport and offered several interpretations. For example, several researchers (Cunningham et al., 2007; Wells and Kerwin, 2016) applied the versatile framework of social cognitive career theory (SCCT) from Lent et al. (1994) to examine the interaction of personal, environmental, and behavioral factors. These researchers, and others, have explored the complexity of a non-traditional, non-linear career paths that women have followed, many tailored to suit their specific needs as well as their career and life goals. More recently, Mainiero and Sullivan (2005) kaleidoscope career model (KCM) has been adopted in sport. Appropriately, Shaw and Leberman (2015) used KCM to frame their research on female New Zealand chief executive officers highlighting their life and career stages, and how their social relationships played out through authenticity, balance, and challenges. Most recently, sport researchers (Hancock and Hums, 2016; Hartzell and Dixon, 2018; Taylor et al., 2018; see Darvin et al., 2019) have begun to acknowledge the non-linear and unpredictable nature of careers in sports and recognize that “individuals must construct an individual career that fits their own lives” (Savickas, 2005, p. 150), so Career Constructive Theory (CCT) has been readily applied.

Though CCT has mainly been used to examine careers in the intercollegiate athletic landscape (see Hancock and Hums, 2016; Hartzell and Dixon, 2018; Taylor et al., 2018; Darvin et al., 2019), CCT was selected for use in the current study as it considers broad societal and cultural contexts with storying telling from the individual's working life. The foundations of CCT include the individual as actor, agent, and author (Savickas, 2005). As an actor, the individual develops an identity from childhood that is shaped by experiences and environments. Then as an agent, the individuals adapt to the tasks, transitions, and trauma in the environment. Finally, as an author, the individual actively creates their own career story pursuing goals, assessing opportunities, and navigating barriers.

### Career Construction Theory

Through the social constructivist lens of Shaw and Leberman CCT (2015), the researchers examined the career experience of women in Canadian professional sport. Although CCT does not predict one's career path, it can be used to understand individuals' interpretation of “… processes, social interaction, and negotiation of meaning” (Savickas, 2005, p. 43). Through the three components of vocational personality, career adaptability, and life themes, CCT was designed to answer “What do people do?” and “Why do they do it?”

According to Savickas (2005), CCT is a theory to explain and predict occupational choice and work adjustment. More closely, the life theme component of CCT helps explain how individuals seek positions that help define their career identity while promoting a sense of social meaning and relatedness. Using sponsorship and mentorship as career advancement tools, candidates can create allies who are positioned to understand their personal abilities, needs, values and interests to help them in all navigate their careers. Another aspect of CCT, know as career adaptability employs the ABC's of career construction and helps mentors and sponsors counsel proteges so they can achieve their goals. The ABCs of career construction help shape the actual problem-solving strategies and coping behaviors that individuals use to synthesize their vocational self-concepts with work roles. The ABCs are grouped into four dimensions of adaptability, namely: concern, control, curiosity, and confidence. Thus, the adaptive individual is conceptualized as (a) becoming concerned about the vocational future, (b) increasing personal control over one's vocational future, (c) displaying curiosity by exploring possible selves and future scenarios, and (d) strengthening the confidence to pursue ones aspirations. Increasing a clients career adaptability is a central goal in the goal of career construction counseling.

#### Vocational Personality

Personal factors form and reinforce “an individual's career-related abilities, needs, values, and interests,” which is referred to as vocational personality (Savickas, 2005, p. 47). In the career development literature, research on personal factors emphasize personality (Wentling, 2003), gender role socialization (Betz and Fitzgerald, 1987), human capital (Burke, 2007), and self-efficacy (Lent et al., 1994). For women in particular, gender roles have affected one's personal values, goals, and self-efficacy (Beatty et al., 2007). Self-efficacy refers to “beliefs in one's capability to organize and execute the courses of action required managing prospective situations” (Bandura, 1997, p. 2). According to Bandura (1997), such judgments of personal capabilities are important because they predict goal setting, perseverance, the outcomes people expect from behaviors, and performance. In seeking to understand why such a disparity of women sport leaders has continued, researchers have pointed to differences between men and women in self-efficacy (Cunningham et al., 2003; Cunningham and Sagas, 2007).

Zenger (2018) suggested that women often have lower confidence levels than men and as a result do not believe that they are prepared for senior leadership roles. In addition, Sartore and Cunningham (2007) proposed that gender roles and stereotypes connected to sport ideologies hinder women in sport organizations, arguing that women might not view themselves as capable leaders due to the existing low levels of power and status of women in society. This results in self-imposed limits on their leadership behaviors (Sartore and Cunningham, 2007). Conversely, Walker et al. (2011) suggested that both male and female collegiate basketball student-athletes exhibited similar confidence and capability to coaching men's college basketball. Most recently, Wells and Kerwin (2016) examined underrepresented individuals, women and racial minorities, in senior sport leadership roles in U.S. collegiate athletics, they uncovered no difference in self efficacy. The biggest barrier for women was determined to be the unfavorable outcomes (i.e., acceptance, respect, fear of failure) that they anticipated after assuming the Athletic Director role.

#### Career Adaptability

Career adaptability is the ability to cope with contextual factors (Savickas, 2005) and development tasks (e.g., new skills and responsibilities). Contextual factors may include social (e.g., mentoring, networking, work-life balance) and structural (e.g., discriminatory hiring practices, stereotyping, opportunity for promotion) determinants. Geiger (2018) reported that women are twice as likely as men to face gender discrimination in the workplace, a finding that might be higher in the traditionally male-dominated sport culture (Harris et al., 2015). An individual's career adaptability recognizes the interplay of skills and abilities, social expectations, vocational interests, perceived opportunities, and peer acceptance of occupational roles. With one's career experience being influence by personal and contextual factors, it is important to understand a person's experiences, perceptions, and responses to such factors.

#### Life Themes

In CCT, the life theme is what *matters* in one's life story (Savickas, 2005). The work individuals do has purpose and their contributions to society matters to other people. Savickas (2005) suggested that life themes guide people to make meaningful choices about their work role. Recognizing occupational selections are not made in isolation (Eccles, 1994), it is important to uncover the meaning of factors (e.g., job transition, personal trauma) influences one's career decisions and development (Savickas, 2005). The pipeline of women gaining relevant career-building experience is sufficiently full in the entry and mid-career stages, but consistent with the findings of several researchers (Pell, 1996; Helfat et al., 2006) women frequently leave organizations at mid-career stage for a host of reasons (e.g., personal priorities, lack of social support) leaving a leaky pipeline at the senior leadership (Hancock and Hums, 2016). While women also leave organizations prematurely when faced with a chilly organizational climate (Hall and Sandler, 1982; Shaw and Hoeber, 2003), they are often overlooked for upper management positions even when qualified (Hoyt and Murphy, 2016). Furthermore, Harris et al. (2015) suggested that women are often discouraged from pursuing more senior leadership roles in the industry by family members and friends. Overall, a women's experience, environment, and critique may limit or facilitate the development of their vocational personality, career adaptability, and life themes.

### Sponsorship and Mentorship as Career Advancement Tools

A mentor provides “guidance focused on professional issues, such as talking about goal setting, pursuing education, and seeking the right experiences to be successful in a position” (Baumgartner and Schneider, 2010, p. 568). A sponsor identifies high-potential individuals and uses his/her platform or position of power to overtly support the individual's career advancement (Foust-Cummings et al., 2011; Hewlett et al., 2011; Dinolfo et al., 2012). Prior to Kram's (1985) mentoring model, the terms mentors and sponsors were used interchangeably in the literature. This changed following a meta-analysis by Friday et al. (2004) and they were the first to delineate the sponsoring and mentoring functions. However, change was slow to progress. Developments in the sponsorship area were slow to emerge (Hewlett et al., 2010). In recent years, the critical role of sponsorship has emerged and become “a distinct, critical, and more powerful phenomenon than traditional mentoring for the career advancement of women” (Bhide and Tootell, 2018, p. 5).

In male-dominated industries, such as sport, mentorship and sponsorship are vital to women, as they are less likely to be selected to top positions, they are less likely to apply for top positions, and they are more likely to experience barriers to career advancement (Shaw, 2006; Bower and Hums, 2013; Travis et al., 2013; Taylor and Wells, 2017). Moreover, engaging with a mentor or sponsor is an effective way for women to not only advance, but to enter a profession (Darvin et al., 2019) and reach upper-echelon leadership positions (Dworkin et al., 2012). Generally, women operate under the premise of a meritocracy believing their hard work will yield advancement; however, it takes more especially when 46% of men, compared to women, are more likely to have a sponsor (Hewlett et al., 2010; Ibarra et al., 2010).

Even with mentorship, Ibarra et al. (2010) revealed women begin their careers behind men and persistently remain behind men. Mentorship helps women understand their operating styles and provides them insight into gaining more opportunities for career success (Ibarra et al., 2010); however, without a sponsor, women are more likely to be unsatisfied with their career progression (Friday et al., 2004; Foust-Cummings et al., 2011). When a woman has someone with high organizational status advocating on her behalf, encouraging her to take an appropriate stretch assignment, women are just as likely as men to get promoted (Ibarra et al., 2010). According to Silva et al. (2012) women are less likely to be selected for “hot jobs” or top positions without a sponsor.

## Methods

A qualitative approach was utilized to explore the career experience, expectations, and recommendations of the women holding Vice-President or higher positions in professional sport in Canada. Throughout the evolving career development literature, theorists (Lent et al., 1994; Savickas, 2005) have recommended qualitative approaches to more effectively identify and understand the dynamic. Specifically, a hermeneutic phenomenological approach was utilized because of the interpretive process of the women's lived experience (Kafle, 2011).

The open-ended questions were developed on the basis of contemporary research in the area. The interview script was assessed for content validity using an expert panel (i.e., three external sport management scholars who lead respected research programs in gender and equity issues in sport leadership). A pilot study with two women who occupied senior leadership roles in the sports industry (but not professional sport) was undertaken in advance of the research to allow the researchers to pre-test their procedures and evaluate the open-ended questions for clarity and relevance. At the conclusion of the pilot study data collection phase, the two women were independently interviewed to ensure that they understood each of the interview questions proposed for use in the study. The pilot study data also allowed the researchers to assess the quality of the data produced, the efficacy of the proposed research procedures (Patton, 2014).

### Participants

The researchers conducted a census of all the female Senior Vice Presidents working within professional sport in Canada (i.e., the Canadian Football League Head Office, the National Hockey League Head Office, the Toronto Blue Jays, the Toronto Raptors, the Vancouver Canucks, the Edmonton Oilers, the Calgary Flames, the Winnipeg Jets, the Toronto Maple Leafs, the Ottawa Senators, the Montréal Canadiens, the British Columbia Lions, the Calgary Stampeders, the Edmonton Eskimos (now Elks), the Saskatchewan Roughriders, the Winnipeg Blue Bombers, the Hamilton TigerCats, the Toronto Argonauts, the Ottawa RedBlacks, the Montréal Alouettes, the Toronto FC, and the Vancouver Whitecaps). The data were collected during the spring months when the schedules of participants would facilitate their participation. The most recent staff directories were reviewed and senior officials were consulted to ensure a valid study frame. At the time of the study, no woman occupied any of the Canadian professional sport Presidents' roles and only seven of the 58 Vice-President roles were filled by women (i.e., 12%). The women occupying these senior leadership roles we all Caucasian, and all had postsecondary educations. Half of the group had professional or graduate degrees. There were no discernable patterns with respect to having an in-house spouse or partner, dependent children, or years of experience with the organization. What they did share was the experience of effectively navigating the system and advancing to the role of vice-president in professional sport in Canada. This qualified them for inclusion in the study.

### Data Collection Procedures

Every female senior vice president (*N* = 7) of a Canadian professional sport team received a letter introducing the study, its objectives, and an invitation to participate in the research project. The participants were advised that their participation was important, but voluntary, and were informed that they would receive a summary paper following the study. A three-step non-response procedure was employed to maximize response rates. The prospective respondents received a reminder phone call if they had not responded within 2 weeks after the pre-study letter was distributed. They received a second phone call reminder if they had not responded after a 3-week time frame. The data collection period was concluded after a 5-week period.

Extensive data were generated through a 45-min semi-structured phone interview that asked respondents to summarize their background and career experiences that helped prepare and propel them into their senior leadership roles. According to Patton (2014), an interview guide is helpful in focusing the interview and help the interviewer make effective use of time. The interviews were designed to produce deeper understandings of the respondents' experience and uncover their suggestions for improving the proportion of women occupying senior leadership roles in professional sport organizations. A copy of the interview guide is presented in Table 1. Each interview was audio recorded to facilitate accurate data capture and analysis. Field notes were also taken by the researchers to ensure accurate data capture.

**Table 1:** Phone Interview Guide for Female Senior Vice Presidents.

Opening Comments: Thank you for agreeing to participate in this study on Women and Leadership within Professional Sport in Canada. This study has been approved by the Research Ethics Review Board (#108676) at Western University. Through this interview, we will gain a better understanding of how women progress to senior leadership positions within Canadian Professional Sport. We have prepared a number of open-ended questions to guide the interview. We are interested in understanding your career experience and your progression to senior leadership position in professional sport in Canada. Please elaborate on each question as much as possible you we can better understand your experience, insights and guidance. We highly value your insights and observations, and as promised, will do all that we can you protect your anonymity. As a token of our appreciation for your participation in the research you will receive a report of the findings. 1. Career Experiences: a. Tell us about your career progression to your current position? b. Are there significant decisions that helped advance your career? c. As you reflect on your career, can you point to critical opportunities that helped you advance your career? d. As you reflect on your career, can you point to any critical mentors who helped advance your career? 2. Perceived Obstacles & Opportunities: a. Did you experience any obstacles or barriers that slowed your career progression? If so, please elaborate. b. Did you enjoy opportunities that you believe helped advance your career progress? If so, elaborate. c. Did you employ any strategies that helped you navigate your career advancement? 3. Suggestions and Advice: a. What advice would you give young women who seek a senior leadership position in professional sport in Canada? b. What early experiences should young women acquire to prepare for, and advance into senior leadership positions in professional sport in Canada. 4. Conclusion: a. Do you have any other comments that would help us understand your experiences and career progression? b. Do you have any final recommendations that would help women progress to senior leadership roles in professional sport in Canada? Thank you

It was critical that the researchers understand the participants' background to help interpret the findings of the research.

### Data Analysis

Pietkiewicz and Smith (2012) recommend a three-step process of analysis for interpretative phenomenological studies: (a) multiple reading and note taking, (b) convert notes into emergent themes, and; (c) seek relationships and cluster themes. Additionally, while developing the final themes it was important to consider the aforementioned research related to women sport leaders and the conceptual considerations. After the interviews were conducted, data were transcribed, and reviewed for accuracy. The researchers initiated the analysis by reading the transcribed interviews in their entirety, so they could better understand the participants' global perspectives. Patton (2014) cautioned researchers about prematurely focusing on the analysis as it may interfere with the openness of naturalistic inquiry and the researchers adhered to his suggestions.

The entire transcription was sent to participants prior to coding and were completely read a second time to allow the researchers to identify the emerging themes and patterns. Finally, the data were incorporated into a mind map to highlight the common and interconnected themes. The researchers identified the central phenomenon, then analyzed and interpreted it by highlighting the common themes. In any phenomenological study, the data must be grouped according to statements that are associated with larger meanings and themes, then followed by an extensive description (Creswell, 2013). The researchers enacted a double-coding process where the data set was coded, and then after a period of time, the researchers returned to the full transcripts and coded the same data set a second time to ensure accuracy (Krefting, 1991). Disagreements between coders, although rare, were discussed until a consensus was reached.

## Results and Discussion

Four themes emerged from these findings of this research, namely for women to: (a) find and become a role model, mentor, and sponsor; (b) create access to networks and opportunities; (c) be self-promoting, and; (d) strategically build a confident career portfolio.

### Find and Become a Role Model, Mentor, and Sponsor

Women interviewed in this study unanimously believed that having role models, mentors, and sponsors were the most important activities that aspiring leaders could undertake if they seek to have careers in senior leadership in sport. This is consistent with the literature in this area (e.g., Eccles, 1994; Helfat et al., 2006; Hancock et al., 2017; Wells and Hancock, 2017). As each participant described their lived experience and their ability to cope with the contextual factors, it became clear that their career progression and advancement were shaped by role models, mentors, and sponsors.

Darvin et al. (2019) research on women assistant coaches in the NCAA confirmed the critical role that role models, mentors, and especially sponsors have on career progression and advancement. Vice President #2 highlighted the importance of others to her career progression when she stated that: “*I have had great leaders and role models in my life who encouraged me, supported me, and opened doors of opportunity for me.”*

Although Vice President #2 specifically referenced the importance of role models, researchers have noted mentors and sponsors provide more direct support. Mentors help prepare colleagues for leadership roles by providing insights and guiding development while sponsors go a step further by advocating, positioning, and risking their reputation for qualified candidates (Ibarra et al., 2010). Aman et al. (2018, p. 152–153) noted that “through social networking and mentoring women role models in sport administration lend their voices to women, show women in decision-making positions, and realistically portray the possibility that women are qualified to be sport leaders.” Women need to see other women in these life roles, and envision themselves occupying a similar role in the future. Role models help build confidence in others to do the same (Gardner and Laskin, 2011). As Billy Jean King clairvoyantly and accurately states, “you have to see it to be it.” Girls and women need female role models as well as female sponsors who are in positions of authority. Others spoke of the role that mentors played in their career advancement. Mentors guidance and support to these women. They worked together to deconstruct situations and discuss strategies that would help the protégé navigate future challenges.

According to Dreher and Cox (1996), mentorship experiences are best when they involve homogenous individuals. However, as noted in this manuscript, few women hold these types of positions in sport, and especially in professional sport, so the purported benefits of sponsorship are often limited for women seeking to advance their careers in the industry. Vice President #5 did not find this to be a limitation. She commented on the excellent male mentors that she had throughout her career and how their coaching and encouragement helped her advance her career. However, Vice President #1 bluntly stated that “*we need more women on the inside to support and advocate for other women*.” Vice President #6 concurred when she proclaimed:

*I have had great mentors during my professional career, but I must admit that the female mentors had to most profound influence on me and my development. I felt that they better understood my situation and the challenges I have, or would face. In addition, they served as a great example for me and spoke on my behalf*.

Having role models (especially female roles models) and mentors are important, but having sponsors was viewed as the most effective strategy for women seeking career advancement (Helfat et al., 2006; Wells and Hancock, 2017). Vice President #1 reinforced this point when she noted that she was “*recruited and endorsed by a woman who worked for the organization she joined. This woman put her neck out for me and upon reflection made a significant difference to me securing the role and advancing my career.”* Sponsors go a step further by using their reputation, influence, and power to advocate for a protégés' and help create opportunity for them (Ibarra et al., 2010; Wells and Hancock, 2017). However, in spite of its impact on career advancement, limited research has been conducted on women strategically using sponsors to advance their careers into leadership roles in professional sport. Each of the Vice Presidents recommended that young women find a sponsor, and preferably a female sponsor. However, due to the relative comparatively few women in senior roles finding supportive male and female mentors and sponsors encouraged. Vice President #6 encouraged women to “*build a strong network who can their network of colleagues who can help the early career women by providing career guidance and opening doors of opportunity. Don't be afraid to ask for their help. It is not a weakness. It is a career-building strategy.”* Sponsors have the potential to give young women an increased sense of confidence and they can open many doors for gaining early leadership experience. These experiences can develop requisite skill sets and teach valuable leadership lessons. Each of the study participants recognized the need for more female role models, mentors, and sponsors, and in many cases used the three terms interchangeably. However, as Vice President #1 noted an advocate who leveraged their position to create opportunities was the differentiator:

*I was lucky. The men and women that I worked with mentored me. They opened doors for me. They introduced me to key people in the industry. They advocated for my work. The sport community can be a closed shop, so this type of networking was very beneficial to me and my career*.

Although Vice President #6 explicitly mentioned “female mentors,” through her description of the mentor's actions who “spoke on my behalf,” the importance of advocacy emerges, which is a role of a sponsor. The study participants pledged to continue supporting aspiring female leaders in sport and they hope that future generations of female leaders will see supporting encouraging other women as a professional obligation. All of the participants live complex and busy lives, but each remained 100% committed to sharing insights that would help those who follow them. They agreed with other writers who suggested that mentors need to “be sensitive to this complexity, and be prepared to help with time management suggestions or tips for overcoming guilt and anxiety” (National Institutes of Health (NIH), 2012, p. 2). For example, Vice President #5 stated:

*There have been times when I was worried that I was spending too much time at the office and neglecting other important parts of my life. Fortunately, I have a great support system to help me. I realize that not everyone has the same support*.

The women interviewed in this study have benefited from positive mentoring and sponsoring and in areas explicitly stated as: (a) improving opportunity and success in career advancement; (b) increasing institutional loyalty; (c) improving time management and productivity behaviors; (d) increasing the procurement of grants; (e) improving mentee satisfaction with professional and work-life balance; (f) instilling higher administrative aspirations in mentees, and; (g) and assisting mentees with improved networking skills. Women need to have access to the perspectives of other women who have forged their way into senior leadership roles in professional sport in Canada. These women are prepared to help. Hopefully, those who follow them will have the same degree of commitment.

Litzky and Greenhaus (2007) suggested that sponsors need to support the next generation of leaders. These Vice Presidents recognized the importance of sponsors to their career advancement and they are committed to “sending the ladder back down” to pick other women up who aspire to senior leadership roles in the industry. These examples of supporting sponsors develop the vocational personality pillar of CCT because sponsors enhance career-related abilities and needs by providing access to projects, jobs, experience, and networks.

### Create Access to Networks and Opportunities

Networking was also identified as one of the most important strategies to assist women advance into senior leadership roles in Canadian professional sport. A number of researchers have documented the impact that networking can have on career advancement (Burt, 1992, 2000; Ibarra, 1997), including success in the sports industry (Burton et al., 2017). This research supported the findings of other researchers (Litzky and Greenhaus, 2007; Sandhu and Mehta, 2008; Wells and Hancock, 2017) who noted that more active and effective networking can make a difference for women seeking senior leadership roles in sport. Vice President #2 reflected that she “*… made career moves that were challenging and developmental, and in hindsight, also helped me [her] expand my [her] network of colleagues and mentors.”* The study participants were united in their belief that the majority of women that they have observed working in the industry do not effectively network. Part of the problem is that women in senior leadership have very few examples of female leaders to network within professional sport in Canada, a reality also expressed in the research of Darvin and Sagas (2017). Greguletz et al. (2018) suggested that women are generally less effective than men in utilizing networks to advance their careers.

Compounding the problem is the fact that researchers have revealed that individuals prefer to function in homogeneous groups (Levine and Moreland, 1990). Although the interaction of homogenous members leads to an increase in social support and information exchange which benefit career outcomes (Cross et al., 2019), it can also impose conformity and exclusion instead of tolerance and acceptance (Blackshaw and Long, 2005), which is what these Vice Presidents are working to create for the next generation. For example, the participants noted that “gendered circumstances” often limited opportunity for networking. Recognizing one's career adaptability is dependent on personal and contextual factors; it is important to understand some women expressed concerns stating that they found networking “hard,” as they were female, and that male networking is often done in settings where some may “feel uncomfortable.” Participants in this study indicated that they overcame this situation by networking outside of professional sport. Vice President #1 advised women to “*get out of their comfort zone and build their experience set and network.”* This strategy could be especially valuable to women seeking female mentors and sponsors given the relative few that exist in professional sport.

Another finding from this research extended the work of Johansen (2007) who concluded that women network differently than their male counterparts. Norman (2010) elaborated on this point by suggesting that networking is often more difficult for women. Sometimes their interests fall outside the work environment. Some women may have childcare responsibilities that preclude afterhours and weekend networking activities. Respondents also noted that participation in this male-focused networking opportunities may have negative professional consequences and as a result, women are often forced to create different ways of networking. Especially in common-pooled resource systems, which exists in sport, demographic differences have created strong divides between individuals (McPherson et al., 2001). Such divides complicate the quality of information received, attitudes formed, and interactions experienced (McPherson et al., 2001). Regardless of the reason, women are not always afforded the same networking opportunities that benefit men and their career development and advancement (Darvin and Sagas, 2017).

Although aspiring female leaders need to form strong and positive female networks, so the group can collaborate and grow together (Litzky and Greenhaus, 2007; Sandhu and Mehta, 2008; Wells and Hancock, 2017), women must also recognize affiliation networks influence the lack of access they have to higher-ranking positions (Katz et al., 2018). The Vice Presidents surveyed in this study pledged to rely on their position of power to help shape a supportive and inclusive workplace, and in doing so, create better opportunities for the next generation of females seeking senior leadership roles in the industry (Ibarra, 1992, 1995). For example, Vice President #6 recommended the creation of a “*Women in Sport Leadership Association.”* She suggested that this group could stage professional development programs, create networking and mentorship opportunities for other women, disseminate helpful and inspiring research literature, and serve as an advocacy voice for current and aspiring females seeking senior leadership roles in sport. The North American Society for Sport Management (NASSM) established the Women in NASSM (WIN) group and it serves as an example of how a group can be helpful in encouraging and supporting colleagues by creating a space for like-minded individuals to gather during conferences, share job opportunities, and disseminate knowledge. Other participants in the study suggested ideas and activities that could also be encompassed by the work of the Association, including being a support network (Vice President #2); creating growth opportunities and professional development (Vice President #3), and offering programs and services that help build confidence for women with leadership aspirations in sport (Vice President #4).

Aspiring female leaders need to connect with other strong female leaders, including those from other industries where sponsorship practices seem to be more prevalent and effective than in the sport domain (Ibarra et al., 2010; Darvin and Sagas, 2017). However, leaders in sport must be mindful of the fact that: “visible institutional support, particularly from senior management, is vital to ensure support for any success of a mentoring program” (Quinn, 2012, p. 14). Institutional support can begin in the form of a mentoring program to create these relationships that may evolve into a sponsorship. Organizations can provide the structure and resources to support an effective mentoring program. Formal programs to prepare mentors and mentees can be developed, and grants are available to support such initiatives (National Institutes of Health (NIH), 2012). Networking is an important strategy to secure a senior leadership position in professional sport (Wells and Hancock, 2017), and developing relationships is critical to advancement in the sports industry (Burton et al., 2017). Through strategic networking, women will increase their exposure and opportunities. However, the study participants were unanimous in their assessment that most women who have, or currently work within the professional sports industry do not effectively network. In addition, the interviewees were united in their perception that in the past, many women that they have worked with lacked valuable self-promotion skills that facilitate access and succeed in these networks. A majority of the study participants identified the need for women to put themselves in situations where they do not feel that they need to be “one of the guys.” They need to feel comfortable showcasing their own skills, talents, and potential. They need to display the confidence and commitment levels comparable to those exhibited by many of their male counterparts.

### Be Self-Promoting

The study participants were unanimous in their claim that women need to stop engaging in self-limiting behaviors. Conversely, they need to take credit for their success. Self-limited behaviors often hold women back from securing senior leadership roles (Zenger, 2018) and consequently, reaching their aspirations as senior leaders. As noted by Vice President #3:

*I put myself out there. I applied for roles that some may have felt were beyond my current level of experience and experience. I was not afraid of rejection. I have noticed that some of the women I mentor fear rejection and therefore don't apply for roles that equally qualified men routinely apply for*.

These behaviors frequently sabotage career advancement for women, especially those seeking senior leadership roles in sport. As Gardner and Laskin (2011) noted, the first step to being a leader is to speak and act like one, similar to the advice offered by Vice President #5 who noted that “I was a varsity athlete and I know what it takes to be part of a successful team striving for group success. The same skill sets have served me well throughout my career.” Additionally, women need to find their voice and promote themselves in a confident manner, consistent with their male counterparts as revealed in Vice President #3's comment that: “*Women need to stand up and take credit for success when merited. I sometimes see women doing the bulk of the work and then shifting to the sidelines when credit is distributed. Be bold and promote yourself.”*

Aman et al. (2018, p. 154) suggested that “… while self-promoting behaviors are rare in women, men use self-promoting behaviors to access leadership positions and gain economic rewards,” so women must do the same (Sandhu and Mehta, 2008). In the male-dominated sport's environment, women often encounter prejudices that impact the perceptions of their effectiveness (Eagly and Karau, 2002; Eagly and Carli, 2007). When women step outside the “expected” gender norm they are often perceived as being too bossy or too assertive (Eagly and Karau, 2002). However, women must confidently promote their skill sets and experience, similar to the manner that their male counterparts employ.

Hall and Sandler (1982) suggested that colleagues can play a significant role in advancing women, and they need to be more inclusive, encouraging, supportive, and helpful in opening doors of opportunity for their female colleagues. That said, women need to put themselves in situations where they do not feel that they need to be “one of the guys.” In particular, several interviewees (Vice Presidents #3, #5, and #6) believe that many women they have worked with lack this instinct. Vice President #2 noted that she has witnessed many women gravitate to clerical roles (e.g., note taking) in Board meetings as opposed to their male counterparts. She suggested that: “*Women need to purposefully showcase their own skills, talents, and experience in senior leadership meetings potential while displaying a level of confidence, commitment, and comfort comparable to the levels that some of their male counterparts exhibit.”* Although we know women in sport are just as confident and capable (Walker et al., 2011; Wells and Kerwin, 2016), Vice President #1 advised women to display their vocational personality by having “*confidence in themselves and their abilities”* and “*stand[ing] up for themselves.”*

### Strategically Build a Confident Career Portfolio

The study participants agreed that working in smaller departments in the early stages of their careers would provide candidates with growth opportunities that they may not garner if they assumed roles in larger institutions. Vice President #5 advised female colleagues to strategically build their careers by focusing on the acquisition of diverse and transferable skill sets, especially early in their careers. She stated that:

*My advice to women seeking senior leadership roles in professional sport is to take positions in and out of sport that will build diverse, but transferable skill sets. I believe that this a strategic way forward for women. Have an end goal in mind, but always take positions based on the learning and networking benefits*.

The recommendation to have women gain multiple industry training is supported in the literature and may help to increase their experience and candidacy for more senior leadership opportunities (Johansen, 2007; Ibarra et al., 2010). Women will learn to take on projects and assume risks early in their career, and in doing so ultimately gaining experience, a track record of success, and the opportunity to bolster their level of confidence. Vice President #4 commented that she “*never made a career move into a position that didn't challenge her.”* She advised women to “*dream big and persevere to make that dream a reality.”*

According to the study participants, women often do not pursue diverse and challenging roles early in their careers that help build their vocational portfolios and experiences. Vice President #5 challenged the next generation to “take leadership of their careers, seek challenging roles that will develop their skills, and build a demonstrated record of achievement. Early roles lay the foundation for senior roles.” Additionally, Vice President #3 confirmed the utility of this strategy when she stated that she: “… applied for roles that some may have felt were beyond my current capabilities and level of experience.” The recommendation to have women gain multiple industry training is well-founded and may increase their career adaptability (Savickas, 2005). Additionally, Vice President #5 suggested that women seize opportunities to heighten their experience levels, develop complimentary skills, and build confidence. She advised women seeking senior leadership roles in professional sport to:

Reach high. Say positive. Be strategic. Take positions that better prepare you for more senior leadership roles. However, never under-estimate the role that your network can have getting you positioned as well as prepared for advancement.

## Conclusion

The researchers analyzed the lived experiences of all women who currently hold Vice President positions in professional sport in Canada. Following Savickas' Career Constructive Theory (2005), these women graciously shared their perspectives and insights that might help other women progress to senior leadership roles in the industry. In doing so, we sought to answer three research questions.

First, we sought to uncover what are the career experiences of women senior leaders. The research revealed real and perceived barriers exist for women senior leaders in professional sport in Canada adding to the growing literature that suggests that although there have been many advances for women, there are still many limitations for those working or aspiring to work at the senior leadership levels of professional sport (Burton, 2015; Burton and Leberman, 2017). Each of the participants in this study experienced varying degrees of challenge that needed to overcome to advance in their professional careers. The respondents noted that they often endured colleagues questioning their leadership abilities, had experienced leaders attempt to stifle their career advancement, and had colleagues who criticized their character and/or leadership behaviors. These experiences align with findings of scholars who study the advancement of women in leadership (Shaw and Hoeber, 2003; Norman, 2010). Although the women in this study reported that they often felt discouraged by the treatment they received, they reported that they found their voice, leaned on trusted colleagues, benefitted from exceptional role models, mentors, and sponsors, and displayed the confidence needed to persevere and advance in the industry. Vice President #3 noted that that she “*… never put barriers on herself and she always trusted her abilities.”* She recommended that women aspiring to these roles to “*… follow their dreams and exhibit leadership in a way that builds your career.”*

Participants all acknowledged the low representation of women within professional Canadian sport and viewed it as a problem. As a result, the candidate pools for senior-level leadership roles tends to be comprised of male colleagues who continued to garner relevant experience and contacts that their female counterparts missed (LaVoi and Dutove, 2012; Hancock and Hums, 2016). Vice President #6 she noted that “*Many women drop out, or choose to remain in mid-career leadership roles in sport. As a result, the candidate pool for senior-level positions is generally 100% men. We have to support and encourage women to stay in the race.”*

The women also commented on the prevalence of self-limiting behaviors that limit upward mobility to senior leadership roles in sport. Although the participants all viewed themselves as capable, they frequently suggested that women they worked with over the course of their careers frequently devalued their competence and contributions. They could recite examples of women who did not pursue leadership positions because they did not feel 100% qualified for the role. Similar to Walker et al. (2011) the participants' believed that the reason women have low self-confidence in leadership roles due to the hegemonic nature of professional sport, not necessarily the individual. It was believed that the combination of barriers and support systems contributed to women not securing leadership positions in proportionate numbers. Vice President #6 encouraged women to “*bite off more than they can chew and have confidence that you will get the job done.”*

Now, the burden cannot all be on the backs of the seven women leaders to advance the next generation of women sport leaders. Men need to be willing to actively participate in employee resource groups (ERGs), mentorship and sponsorship of women (Valerio and Sawyer, 2016; Johnson and Smith, 2018, 2020). Recognizing the unique life themes women may experience, similar to Shaw and Leberman (2015), the conceptual framework should be adapted to reflect the complexities of women's experiences. While all of the participants shined a light on what they, as women, had to do to succeed, and encouraged other women to persistently navigate, maneuver, and change, this revelation also speaks to the restrictive nature of current systems, gatekeepers, policies, and practices.

Relying on individual agency alone will not advance the next generation of women sport leaders, both elements of personal agency and organizational structure are necessary. By understanding the complexity of the issues facing women, sport organizations can ensure that the talents and experiences of strong and effective female leaders are not lost. Current leaders and team owners, majority of whom are men, must encourage, create, and implement supportive and inclusive policies and practices that promote equality in senior leadership positions within professional sport in Canada. It is recommended that researchers continue to study this issue, and sport organizations implement the findings of this research, so needed change can transpire. One ideal way to study this would be to conduct a longitudinal study with a cohort of entry-level women in working in professional sport in Canada. By following the career progression over time, we can examine the experience over a broader context within the multi-levels of individuals, organizations, and society.

Secondly, we researched how, if at all, were women senior leaders supported in their pursuit of career advancement? We discovered that the women who persevered and advanced to senior leadership roles in professional sport in Canada all reported having role models, mentors, and especially sponsors who were critical to their success. These women underscored the importance of having and strategically utilizing the support and influence of role models, mentors, and sponsors. Sponsors who brought opportunities to their attention and then used their influence and reputation to advocate for others were especially helpful to these women. Having role models and working close with mentors (e.g., for coaching/advice) and sponsors (who will advocate for them) was critical for these women, and was viewed as an excellent strategy for women wishing to follow them into senior leadership roles in the industry. While it would be ideal to have women serve in these mentor and sponsor roles, the relative low numbers may pose a challenge at this point in time. These women indicated that men often filled these roles for them, but they were confident that the situation would change in the future. The Vice Presidents unanimously indicated that they would be happy to serve in these capacities for those wishing to follow their career paths.

Finally, we evaluated what these female senior leaders recommended to the next generation of women who seek senior roles in the industry. The Vice Presidents once again pointed to the lack of female role models, mentors and especially sponsors who could provide valuable guidance, advocacy, and help foster opportunity for future generations of female leaders in sport. They also highlighted the need for progressive human resource policies might keep women in the pipeline long enough to build their experience and confidence, and better prepare them for senior leadership opportunities when they come available. If so, the cycle could be broken, and these women could join others in serving as role models, mentors, and sponsors to future generations of female leaders in professional sport.

The Vice Presidents acknowledged that the next generation of women need to create different ways of effectively networking. Applying CCT, an individual's career-related abilities and needs can be enhanced by being connected into the right networks, particularly if connected via a sponsor.

By learning problem-solving strategies and coping behaviors from a sponsor, an adaptive individual who may have concerns about their career can increase their personal control and strengthen their confidence (Savickas, 2005). Furthermore, respondents noted that participation in this male-focused networking opportunities worked for them; however, as the Vice Presidents reflected, they now see the vital role of also having female advocates and roles models specifically because of their lived experiences as a woman. Through the lens of CCT's life theme, knowing the hardships, and possible trauma, women experience endlessly navigated the male-dominated industry, the next generation of women leaders can create a sense of relatedness and social identity to other women working in the industry. Finally, all of the Vice Presidents' career stories revealed how meaningful their work is for not only them personally, but for the success of the next generation. With conviction they suggested that many women need to find their voice and promote themselves in a confident manner, consistent with their male counterparts as revealed in Vice President #3's comment that: “*Women need to stand up and take credit for success when merited. I sometimes see women doing the bulk of the work and then shifting to the sidelines when credit is distributed. Be bold and promote yourself.”* To make economic gains and access more leadership positions, women must use self-promoting behaviors (Sandhu and Mehta, 2008).

## Implications and Future Research

Theoretical and practical implications manifested as all the women working in vice president positions of professional sport teams in Canada recounted their lived experiences and offered advice to the next generation. One of the main contributions to the sparse sport career development research that has historically been focused on the context of American collegiate athletics was the professional sport context in Canada. Based on the career trajectory of all the women vice presidents, our research extended the sponsorship literature in sport (Darvin et al., 2019) and revealed how the intricacies of career construction extend beyond one event or experience (Savickas, 2005).

Sports organizations must foster a workplace culture that is supportive of women who aspire to senior leadership positions. Sponsors and mentors also need to be acknowledged and rewarded by their organizations. Sport organizations need to create an environment that supports and rewards leadership for both men and women. “Recognition of sponsorship is likely to increase the number of senior leaders willing to spend time sponsoring more junior colleagues” (Block and Tietjen-Smith, 2016, p. 310). Weiner and Burton (2016) noted that both men and women view effective leadership as having a communal orientation through reinforcing the importance of workplace collaboration, capacity building and shared decision-making. These elements are the tenets of current thinking in leadership (Weese, 2018) and according to Gerzema and D'Antonio (2013) and Deane et al. (2015), women perform better than men in these critical leadership areas. For example, women and men are indistinguishable in other key leadership areas (e.g., intelligence, innovation), however, women are stronger in critical areas like compassion and organizational skills. The same research base confirms that women, compared to men, are perceived as being more honest and trustworthy, attributes that are also critical to leadership success (Weese, 2018).

Professional sport organizations would be well-served in employing higher proportions of women in senior leadership roles. A vast amount of leadership talent is defecting, and a significant demographic is being overlooked. Why would any organization willfully neglect 50% of the population, especially when research suggests superior results from result from a more inclusive leadership group (Desvaux et al., 2007; Zenger and Folkman, 2012; Young, 2015). Change may be coming according to some, but according to the study participants and the women in sport leadership literature base, it is unfolding at a snail's pace, all in spite of affirmative action laws and employment equity legislation (Lough and Grappendorf, 2007). The male-dominate and prominent sports domain may warrant heightened affirmative action to make significant change (Burton, 2015) to break the cycle and create more female role models, mentors, and sponsors for the next generation of women interested in a career in senior leadership in sport. Vice President #1 reminded us that “*We are making progress, but not at the rate we should. However, I am encouraged by the inclusive and supportive leadership practices that I am now seeing from corporate boards, hiring committees, and senior leaders.”* Let's hope that significant and sustainable change can happen in an expedited manner.

## Data Availability Statement

The datasets presented in this article are not readily available because Respondents were assured of their anonymity. Requests to access the datasets should be directed to Janelle E. Wells, jweese1@uwo.ca.

## Ethics Statement

The studies involving human participants were reviewed and approved by Western University Research Ethics Board. The patients/participants provided their written informed consent to participate in this study.

## Author Contributions

All authors listed have made a substantial, direct and intellectual contribution to the work, and approved it for publication.

## Funding

This work was supported by Internal Research Support from the Provost's Office.

## Conflict of Interest

The authors declare that the research was conducted in the absence of any commercial or financial relationships that could be construed as a potential conflict of interest.

## Publisher's Note

All claims expressed in this article are solely those of the authors and do not necessarily represent those of their affiliated organizations, or those of the publisher, the editors and the reviewers. Any product that may be evaluated in this article, or claim that may be made by its manufacturer, is not guaranteed or endorsed by the publisher.
